# Supplementary material for: Development of a computer-aided design software for the quantitative evaluation of aesthetic damage
Source: PLoS One. 2019 Dec 18;14(12):e0226322. doi: 10.1371/journal.pone.0226322 (PMC6919621; doi:10.1371/journal.pone.0226322)
Supplement: S1 Code site — 1. (ZIP) [file pone.0226322.s001.zip › [EESC_jr][Projeto]Relatorio_07_21/reports/templates_zip/gentelella-master.zip!/gentelella-master/production/profile.html]

Gentellela Alela! | 


Gentellela Alela!

Welcome,

## John Doe

  


### General

- Home 
  - Dashboard
  - Dashboard2
  - Dashboard3
- Forms 
  - General Form
  - Advanced Components
  - Form Validation
  - Form Wizard
  - Form Upload
  - Form Buttons
- UI Elements 
  - General Elements
  - Media Gallery
  - Typography
  - Icons
  - Glyphicons
  - Widgets
  - Invoice
  - Inbox
  - Calendar
- Tables 
  - Tables
  - Table Dynamic
- Data Presentation 
  - Chart JS
  - Chart JS2
  - Moris JS
  - ECharts
  - Other Charts
- Layouts 
  - Fixed Sidebar
  - Fixed Footer

### Live On

- Additional Pages 
  - E-commerce
  - Projects
  - Project Detail
  - Contacts
  - Profile
- Extras 
  - 403 Error
  - 404 Error
  - 500 Error
  - Plain Page
  - Login Page
  - Pricing Tables
- Multilevel Menu 
  - Level One- Level One
      - Level Two
      - Level Two
      - Level Two
    - Level One
- Landing Page Coming Soon

- John Doe
  - Profile
  - 50%
    Settings
  - Help
  - Log Out
- 6
  - John Smith
    3 mins ago

    Film festivals used to be do-or-die moments for movie makers. They were where...
  - John Smith
    3 mins ago

    Film festivals used to be do-or-die moments for movie makers. They were where...
  - John Smith
    3 mins ago

    Film festivals used to be do-or-die moments for movie makers. They were where...
  - John Smith
    3 mins ago

    Film festivals used to be do-or-die moments for movie makers. They were where...
  - **See All Alerts**


### User Profile

Go!

## User Report Activity report

- - Settings 1
  - Settings 2

### Samuel Doe

- San Francisco, California, USA
- Software Engineer
- www.kimlabs.com

Edit Profile
  


#### Skills

- Web Applications
- Website Design
- Automation & Testing
- UI / UX

## User Activity Report

December 30, 2014 - January 28, 2015

- Recent Activity
- Projects Worked on
- Profile

- ### 24

  May

  #### Desmond Davison

  > Raw denim you probably haven't heard of them jean shorts Austin. Nesciunt tofu stumptown aliqua butcher retro keffiyeh dreamcatcher synth.

    

  User Acceptance Test.doc
- ### 21

  May

  #### Brian Michaels

  > Raw denim you probably haven't heard of them jean shorts Austin. Nesciunt tofu stumptown aliqua butcher retro keffiyeh dreamcatcher synth.

    

  Download
- ### 24

  May

  #### Desmond Davison

  > Raw denim you probably haven't heard of them jean shorts Austin. Nesciunt tofu stumptown aliqua butcher retro keffiyeh dreamcatcher synth.

    

  User Acceptance Test.doc
- ### 21

  May

  #### Brian Michaels

  > Raw denim you probably haven't heard of them jean shorts Austin. Nesciunt tofu stumptown aliqua butcher retro keffiyeh dreamcatcher synth.

    

  Download

| # | Project Name | Client Company | Hours Spent | Contribution |
| --- | --- | --- | --- | --- |
| 1 | New Company Takeover Review | Deveint Inc | 18 |  |
| 2 | New Partner Contracts Consultanci | Deveint Inc | 13 |  |
| 3 | Partners and Inverstors report | Deveint Inc | 30 |  |
| 4 | New Company Takeover Review | Deveint Inc | 28 |  |

xxFood truck fixie locavore, accusamus mcsweeney's marfa nulla single-origin coffee squid. Exercitation +1 labore velit, blog sartorial PBR leggings next level wes anderson artisan four loko farm-to-table craft beer twee. Qui
photo booth letterpress, commodo enim craft beer mlkshk


Gentelella - Bootstrap Admin Template by Colorlib
